# Multi-domain computerized cognitive training program improves performance of bookkeeping tasks: a matched-sampling active-controlled trial

**DOI:** 10.3389/fpsyg.2014.00794

**Published:** 2014-07-28

**Authors:** Amit Lampit, Claus Ebster, Michael Valenzuela

**Affiliations:** ^1^Regenerative Neuroscience Group, Brain and Mind Research Institute, University of SydneySydney, NSW, Australia; ^2^Lauder Business SchoolVienna, Austria; ^3^Department of Marketing, University of ViennaVienna, Austria

**Keywords:** cognitive training, bookkeeping, young adults, job performance, far transfer

## Abstract

Cognitive skills are important predictors of job performance, but the extent to which computerized cognitive training (CCT) can improve job performance in healthy adults is unclear. We report, for the first time, that a CCT program aimed at attention, memory, reasoning and visuo-spatial abilities can enhance productivity in healthy younger adults on bookkeeping tasks with high relevance to real-world job performance. 44 business students (77.3% female, mean age 21.4 ± 2.6 years) were assigned to either (a) 20 h of CCT, or (b) 20 h of computerized arithmetic training (active control) by a matched sampling procedure. Both interventions were conducted over a period of 6 weeks, 3–4 1-h sessions per week. Transfer of skills to performance on a 60-min paper-based bookkeeping task was measured at three time points—baseline, after 10 h and after 20 h of training. Repeated measures ANOVA found a significant Group X Time effect on productivity (*F* = 7.033, *df* = 1.745; 73.273, *p* = 0.003) with a significant interaction at both the 10-h (Relative Cohen's effect size = 0.38, *p* = 0.014) and 20-h time points (Relative Cohen's effect size = 0.40, *p* = 0.003). No significant effects were found on accuracy or on Conners' Continuous Performance Test, a measure of sustained attention. The results are discussed in reference to previous findings on the relationship between brain plasticity and job performance. Generalization of results requires further study.

## Introduction

Cognitive abilities are one of the most significant predictors of future job performance (Schmidt, 2002). The question therefore arises as to whether interventions that augment cognitive and psychomotor skills can improve work-related outcomes (Arthur et al., 2003; Academy of Medical Sciences, 2012). Computerized cognitive training (CCT), which is particularly effective in clinical groups and the elderly, is one method for improving cognitive performance (Buschert et al., 2010; Vinogradov et al., 2012). A recent report by leading British scientific societies cited CCT as a potentially effective intervention to improve workplace performance, stressing the importance of examining the transferability of CCT into vocational tasks (Academy of Medical Sciences, 2012). Previous studies have reported positive effects of CCT on tasks with high psychomotor demands such as flight (Hart and Battiste, 1992; Gopher et al., 1994), driving (Roenker et al., 2003; Cassavaugh and Kramer, 2009; Pradhan et al., 2011) and laparoscopic surgery (Schlickum et al., 2009; Adams et al., 2012), as well as enhanced employment outcomes in schizophrenic patients (Vauth et al., 2005b; McGurk et al., 2007; Bell et al., 2008; Lindenmayer et al., 2008; McGurk et al., 2009). Conversely, a recent RCT conducted by our group (Borness et al., 2013) did not find any effect of an online CCT program on job performance in a large cohort of white collar employees of an Australian public sector organization. Thus, the extent to which CCT can augment performance of middle-skill office tasks in young healthy adults remains unclear. Here, we use a classic example of mid-level skilled occupational task—bookkeeping—to explore the effectiveness of a cognitive training program on work-related task performance.

The rapid computerization of accounting practice has transformed the nature of contemporary bookkeeping into repetitive translation of transactions from natural language into an accountancy software system (Bhaskar et al., 1983; Cooper and Taylor, 2000). The objective of basic bookkeeping tasks is to analyze and organize newly acquired data according to the principles of double entry bookkeeping and subsequently transpose these into journal entries. The objectivity of these tasks, their prevalence in accounting education, and their relatively low dependency on external factors (e.g., trends in consumer behavior) make bookkeeping a convenient and occupationally- relevant work-related outcome when studying skill acquisition and the effect of cognitive factors (Dillard et al., 1982).

An accurate entry of transactions into an accounting system is a bookkeepers' key performance indicator; therefore, our measure of productivity was based on the number of transactions correctly transposed. Both speed and accuracy of work are important, as speed (transactions per unit of time) determines the maximum number of transactions that a worker can execute while accuracy rate (the ratio of correct entries out of total transactions) reflects the relative frequency of errors. Theoretically, improvements in productivity could result from an increase in speed and/or accuracy. However, in practice, increases in task speed usually decrease accuracy, a phenomenon known as the speed-accuracy tradeoff (Wickelgren, 1977). When evaluating the effectiveness of any CCT program on work-related productivity, the potential influence of such tradeoff must therefore be analyzed (Förster et al., 2003).

Since a CCT intervention typically involves many procedural features beyond the CCT program itself, which include trainer contact, socialization, motivational prompts, increased stimulation, and the traditional Hawthorne expectancy bias and retest effect, it is vital to compare the putative effects of CCT on productivity outcomes with respect to an intensity-matched active control (AC) condition to make valid inferences (Jacoby and Ahissar, 2013). Yet, of the thirteen trials of CCT in vocational settings mentioned above, seven (Gopher et al., 1994; Vauth et al., 2005a; McGurk et al., 2007, 2009; Bell et al., 2008; Cassavaugh and Kramer, 2009; Pradhan et al., 2011) did not include an AC arm. We therefore aimed to assess whether CCT can increase the speed or accuracy of work-related task productivity over and above any effects seen in an active control condition.

## Materials and methods

### Research design

This study was a matched-sampling, double-blind, repeated-measures, active-control trial. Subjects were allocated to either (1) 20 h of CCT arm or (2) active control arm matched in time and intensity. Performance on a 60-min paper-based bookkeeping task (see below) was measured three times, at baseline, after 10 h of training, and after 20 h of training. All procedures took place in a university classroom converted into a training lab. The study was approved by the Lauder Business School Ethics Committee.

### Participants and sampling procedure

In January 2009, all at Lauder Business School (*n* = 242), an English-language teaching business school in Vienna, Austria, were invited by email to participate in the study. None of the participants spoke English as first language. Inclusion criteria were (1) successful completion of at least one semester in accounting, (2) lack of any major neurological or psychiatric disorders, and (3) active participation in a scholarship program offered by the school. These criteria ensured an adequate understanding of basic accounting principles, including the posting task used in this study, and enabled compensation to be offered to volunteers as outlined below. All candidates gave a written informed consent prior to the baseline assessment.

Of the 44 participants who completed the study, 34 (77.3%) were females and 10 were males. Four subjects (9%) were left-handed. The average age was 21.4 ± 2.6 years. Fourteen participants (31.8%) were 2nd semester students, 15 (34.1%) were 4th semester students, 14 (31.8%) were 6th semester students, and 1 (2.3%) was an 8th semester student. Subjects were compensated with one “credit” (which provides approximately €40 worth of dining and housing from the scholarship program) for every hour of participation. The total number of credits was 25, equivalent to about €1000, provided in-kind by the school. Compensation was offered for participation (in hours) rather than on a pay-per-performance basis. To ensure ethical compensation, participants who left the study prematurely were compensated based on the number of hours performed.

Due to the conservable attentional demand of CCT and documented interactions between baseline cognitive ability and response to CCT (White and Shah, 2006), we aimed to distribute attentional skill equally between our two groups. The Conners' Continuous Performance Test (Conners, 2000) was administered to assess sustained attention. Two scores derived from the test, namely confidence interval and response time variability (Response Time Block Change, RTBC), were used. A lower confidence interval indicates better overall attention skills, as does RTBC as it approaches zero (Homack and Riccio, 2006). Subjects with the same confidence interval were graded according to the RTBC score and then divided into pairs.

### Measures

#### Primary outcome measure: performance on a bookkeeping task

Performance on a repetitive paper-based posting task was the primary outcome measure. The task was based on basic rules of double-entry bookkeeping (Bhaskar et al., 1983) and adapted from didactic tasks included within the students' curriculum (Kieso et al., 2004). Task materials included: (1) A journal of 300 randomly generated transactions, based on 25 different cash and accruals transactions (i.e., payables and receivables) with a sum between 1 and 500; (2) A blank general ledger containing 14 accounts (assets, liability, equity, income, and expenses), with a summary line every 37 lines of data; and (3) An instruction sheet containing the correct debit and credit postings for each of the 25 types of transactions, which was designed to help participants when not sure how to handle a transaction. Examples of the materials used for the measuring bookkeeping performance are provided in the Supplementary Material.

Three 60-min bookkeeping test sessions were administered, at baseline (pre-training, T1), after 10 h of training (T2) and after 20 h of training (T3). Every test session started with 3 practice tasks to unsure understanding. Participants were asked to work as quickly and as accurately as possible. A performance goal of 200 transactions per hour (based on a pilot study) was defined to facilitate self-regulation (Kozlowski and Bell, 2006). A blinded research assistant recoded the number of correct debit and credit postings entries in the corresponding row according to the rules provided (productivity) and total entries (to calculate accuracy rate). Only rows in which both postings (debit and credit) were entered according to the rules were considered as correct.

#### Secondary measure: sustained attention

In addition to its role in the matched-sampling process, the Conners' Continuous Performance Test (Conners, 2000) was used to detect potential intervention effects on participants' attention skills. The test was administered at baseline and post-training. The confidence interval, RTBC and Overall (i.e., average) Hit Reaction Time measures were used (with a lower score indicating better performance).

### Interventions

Both the experimental (CCT) and active control (AC) groups participated in 3–4 1-h training sessions weekly for the duration of 6 weeks—the total duration of training in both groups was 20 h. Supervised training took place in a computer lab with up to six subjects per session. Supervision aimed to enhance motivation and meta-cognition, according to studies of instruction in cognitive training (Gopher et al., 1994; Salas and Burke, 2002; Sandford, 2003; Leemkuil and De Hoog, 2005). The training software played sounds through standard speakers, producing a degree of auditory distractions for all participants and increasing selective attentional demands.

#### Computerized cognitive training (CCT)

CCT was based on the commercially available cognitive training software “Captain's Log” (BrainTrain Inc, Richmond, VA). Captain's Log has a history of use in clinical populations such as adults diagnosed with traumatic brain injury (Tinius and Tinius, 2000; Stathopoulou and Lubar, 2004), schizophrenia (Bellucci et al., 2003), and chronic psychiatric disorders (Burda et al., 1994) as well as children with attention difficulties (Rabiner et al., 2010) and older adults (Eckroth-Bucher and Siberski, 2009).

An adaptive and personalized training program was set using the software's Personal Trainer Wizard, which adjusts training difficulty and content according to predefined settings as well as individual performance. Visual and auditory distractions were set at 10% of cases for each type. Seven exercises from the Conceptual Memory Skills module (The Ugly Duckling, Happy Trails, Total Recall, Domino Dynamite, Tower Power, Max's Match, and What's Next) and five exercises from the Numerical Concepts/Memory Skills modules (Bits and Pieces, Match Maker, City Lights, Counting Critters, and Happy Hunter) were used. Table 1 describes the cognitive skills trained by each exercise. A detailed description of each of the exercises is provided in the Supplementary Material.

**Table 1:** **Cognitive skills trained by exercise**.

**Exercise**	**Skills trained**													
The Ugly Duckling	CPS	CR				VP			WM				IM	
Happy Trails		CR			VSQ		VPS		WM				IM	SLA
Total Recall		CR		VSC			VPS		WM			GA	IM	
Domino Dynamite		CR	DA		VSQ		VPS						IM	
Tower Power		CR			VSQ	VP		VT		FMC				SLA
Max's Match	CPS	CR		VSC								GA	IM	
What's Next	CPS	CR	DA	VSC	VSQ				WM		FA		IM	
Bits & Pieces	CPS	CR		VSC		VP			WM			GA	IM	
Match Maker	AA	CR				VP							IM	SLA
City Lights		CR		VSC					WM			GA	IM	
Counting Critters		CR		VSC	VSQ	VP			WM			GA	IM	
Happy Hunter		CR			VSQ				WM		FA		IM	

*AA, Alternating Attention; CPS, Central Processing Speed; CR, Conceptual Reasoning; DA, Divided Attention; FA, Focused Attention; FMC, Fine Motor Control; GA, General Attention; IM, Immediate Memory; SLA, Selective Attention; VP, Visual Perception; VPS, Visual Processing Speed; VS, Visual Scanning; VSC, Visuospatial Classification; VSQ, Visuospatial Sequencing; VT, Visual Tracking; WM, Working Memory. Detailed description of the exercises can be found in the Supplementary Material*.

#### Active control (AC)

Several studies suggest that accounting is associated with strong arithmetic skills, a view that is more likely based on accountancy stereotypes rather than actual demands of the profession (Wells and Fieger, 2006). We therefore chose arithmetic training as our AC condition to control fully for non-specific training effects as well as maintenance of blinding of subjects to the training condition of interest. AC training was based on Maths Trainer, a commercially available computerized arithmetic training program (Oak Systems, Binstead, UK). The program entails 24 different arithmetic exercises (addition, subtraction, multiplication, division, fractions, percentage, number sequence, rounding), as well as daily tests, kakuro and sudoku. The level of complexity rises as the user progress along the training program. Since a typical training session on Maths Trainer lasts about 45 min, subjects in the AC group also played Math Ninja (a freeware published by Piotr J. Walczack) a computerized calculation game, for about 10 more minutes during each session in order to match the duration of CCT sessions.

### Analysis

Analyses were perfomed using SPSS 20. Time X Group effects were tested using repeated measures ANOVA. Where assumption of sphericity was violated, the Greenhouse-Geisser correction was applied to degrees of freedom. Within-group and relative effect sizes were calculated using Cohen's ds at T2 and T3, using difference from baseline and pooled standard deviation for each time point.

## Results

### Recruitment and participant flow

**Figure 1:**
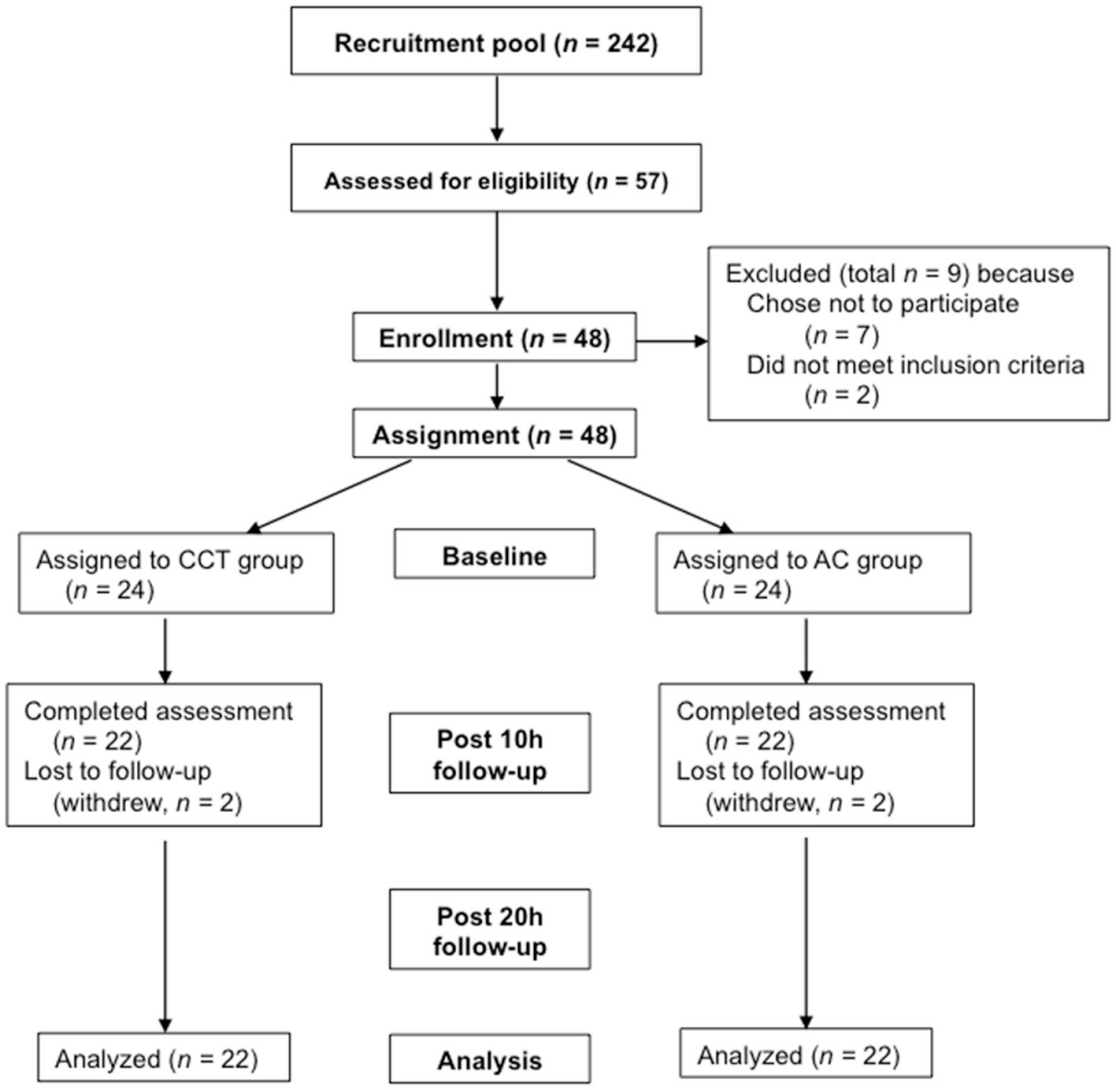
**Study design and participants flow**.

Participant flow is depicted in Figure 1. Overall, 62 students expressed interest to participate. 57 of them were screened for eligibility and were administered the baseline Continuous Performance Test; 48 attended an introductory lecture and completed baseline bookkeeping assessment. Subsequently, they were assigned to groups. Forty four completed the first and second follow-up sessions. Of the four participants who left the study following group assignment (two from each group), three withdrew from the study and one was expelled from the university for reasons unrelated to the study.

### Baseline data

Descriptive statistics and baseline performance are provided in Table 2. Two-sample tests revealed no significant differences between the treatment groups in age, gender, Continuous Performance Test scores, productivity, and accuracy. Pearson zero-order intercorrelations analysis among the study variables at baseline found a positive correlation between age and attentional capacity, as indicated by lower Continuous Performance Test confidence interval, which implies lower probability of attention disorders (*r* = −0.305, *p* < 0.05). Males performed worse than did females on this measure (*T* = −2.82, *p* = 0.007). Students who had completed more years of their university degree also performed better in baseline bookkeeping (*r* = 0.446, *p* < 0.01). There was no relationship between Continuous Performance Test scores and bookkeeping performance.

**Table 2:** **Baseline measures**.

	**Cognitive training**		**Active control**		***p***
	**(*n* = 22)**		**(*n* = 22)**		
	***M***	***SD***	***M***	***SD***	
1. Sex^a^	72.7		81.8		0.47^b^
2. Age	21.27	2.51	22.0	2.71	0.36
3. Completed university years	1.27	1.16	1.64	1.18	0.31
4. Productivity^c^	143.09	50.4	128.91	41.57	0.31
5. Accuracy (%)	91.65	11.71	89.38	7.05	0.44
6. CCPT-II CI	27.07	14.75	28.25	15.69	0.80
7. CCPT-II RTBC	−0.021	0.09	−0.02	0.05	0.64
8. CCPT-II Hit RT (msec)	346.46	49.27	352.79	54.07	0.69

a *Percentage of females*.

b *Pearson X^2^*.

c *No. of correct entries in a 60-min session*.

*CI, Confidence index; RTBS, Response time block change*.

### Primary outcomes

**Figure 2:**
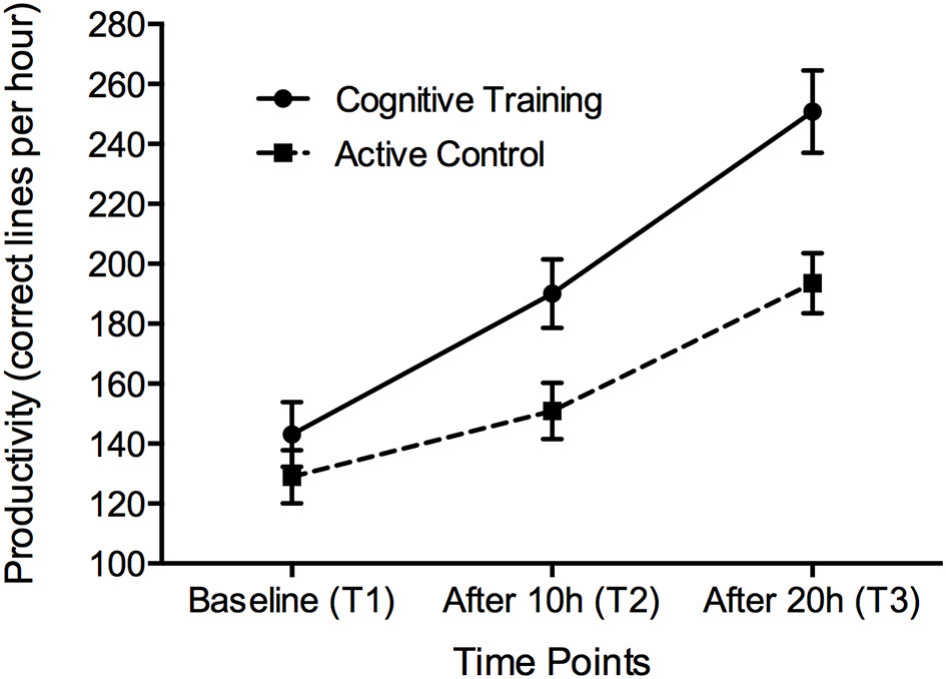
**Performance on the 60-min bookkeeping task at the three time points**. Error bars represent standard error. Significant TIME X GROUP differences were observed at T2 and T3.

Table 3 reports changes in the outcome variables across the three time points. Repeated measures ANOVA found a significant Group X Time interaction effect on productivity, as defined by the number of correct entries in a 60-min test session (*F* = 7.033, *df* = 1.745; 73.273, *p* = 0.003), at both the 10-h (Cohen's effect size = 0.38, *p* = 0.014) and 20-h time points (Cohen's effect size = 0.40, *p* = 0.003—see Figure 2). No significant effects were found for accuracy, although slight improvements of accuracy rates were noted in both the CCT and the AC group (2.61 and 4.69%, respectively).

**Table 3:** **Estimated marginal means, change rates and summary statistics for outcome variables by training group and assessment time**.

	**Cognitive training**			**Active control**			**Group × Time**		
	**(*n* = 22)**			**(*n* = 22)**			**Relative effect size**		
	***M***	***SD***	**Change, %^a^**	***M***	***SD***	**Change, %^a^**	***F***	***p***	***d***
**Productivity, T2**	**190**	**53.88**	**32.78**	**150.91**	**43.91**	**17.07**	**6.521**	**0.014**	**0.38**
Accuracy, T2 (%)	95.58	2.97	4.29	93.24	3.33	4.32	0.001	0.983	−0.24
**Productivity, T3**	**250.73**	**64.65**	**75.23**	**193.5**	**46.98**	**50.1**	**10.226**	**0.003**	**0.40**
Accuracy, T3 (%)	94.32	4.35	2.91	93.57	3.59	4.69	0.225	0.638	−0.45
CCPT CI, T3	21.77	13.15	−19.61^b^	26.1	17.21	−7.61^b^	3.665	0.62	0.25
CCPT RTBC, T3^c^	−0.0005	0.018	−97.62	0.0064	0.016	−1.64	0.663	0.42	0.13
CCPT Hit RT, T3	352.79	26.4	3.37	332.35	56.44	−5.79	0.527	0.47	−0.07

a *From baseline*.

b *Negative scores mean improvement*.

c *Scores closer to zero represent higher attentional performance*.

*The results in bold highlight significant TIME X GROUP interactions. CCPT, Continuous Performance Test; CI, Confidence index; RTBS, Response time block change; RT, Response time*.

The results indicated strong correlations between performance in these two time points for both productivity (*r* = 0.65, *p* < 0.01) and accuracy (*r* = 0.707, *p* < 0.01). Whilst the speed of correct entries increased in the CCT group as a result of our training (i.e., increased productivity), we observed no evidence for a corresponding drop in accuracy, as accuracy rates at baseline (91.65%) were similar to accuracy at both T2 (95.58%) and T3 (94.32%) (repeated measures *F* = 0.225, *p* = 0.638).

### Secondary outcomes

The results indicated no significant TIME X GROUP interaction on either Continuous Performance Test Confidence Index (*F* = 3.665, *p* = 0.62), Response Time Block Change (*F* = 0.663, *p* = 0.62) or Overall Hit Reaction Time (*F* = 0.527, *p* = 0.47, see Table 3).

## Discussion

Participation in 10 and 20 h of CCT produced significant relative Cohen's effect sizes (i.e., after accounting for active control training effects) of 0.38 and 0.40 on an untrained bookkeeping task. Because the active control condition included all non-specific factors inherent to training, such as socialization, supervisor interaction, motivational factors, as well as to some extent cognitive stimulation, the observed effects are unique to CCT and cannot be explained by test-retest and Hawthorn effects. Moreover, since performance on similar bookkeeping tasks is generally predictive of actual bookkeepers' proficiency (Bhaskar et al., 1983), it is reasonable to hypothesize that this outcome may potentially generalize into real-life job performance in the bookkeeping sector. As CCT may represent a cost-effective type of on-the-job training for the better promotion of workforce productivity (Academy of Medical Sciences, 2012), replication of this study in the workplace environment is critical.

We sought to determine whether changes in measures of attention accompanied and potentially explained our observed bookkeeping effects. None of the Continuous Performance Test measures were correlated with either baseline bookkeeping performance or changes thereof, and CCT produced no discernible change in any of the test measures. One interpretation of this result is that sustained attention and inhibitory control has no ecological relevance to bookkeeping task performance in healthy young adults. Alternatively, a more likely explanation for the lack of effect on response inhibition is that our cognitive training program did not specifically target this domain, and so the resulting improvement in bookkeeping skills may be attributed to other cognitive domains. Similarly, we did not observe any difference in the Continuous Performance Test's response time measure, whilst participants in both groups clearly improved their information processing speed ability on the bookkeeping task.

Therefore, whilst the results indicate that CCT can enhance performance on the target bookkeeping task, the nature of the underlying cognitive mediators remain unclear. Lack of additional cognitive measures to probe for these mechanisms is therefore a major limitation of this study, and future studies should complement functional outcomes with a wider neuropsychological battery. What cognitive changes could theoretically mediate the observed improvement in bookkeeping performance? Previous task analyses in the field of bookkeeping (Dillard et al., 1982; Bhaskar et al., 1983; Dillard, 1984) point to several cognitive processes that may be taxed by bookkeeping tasks and thus underpin skill acquisition. Typically, bookkeeping involves integrating new information (a particular transaction) with previous knowledge of how to categorize this information within the bookkeeping set of accounts. Working memory and rule-based decision-making may therefore play an important role in bookkeepers' ability to quickly and accurately classify and record transactions (Dillard, 1984). It is also clear that participants in the CCT group spent less time on each individual transaction compared to controls, suggesting a CCT-induced improvement in information processing speed. Indeed, of the 12 exercises in our CCT regimen, 8 (66%) had a working memory component, 7 (58%) had a processing speed component, and all had a conceptual reasoning component at increasing rate of difficulty (see Table 1 and Supplementary Material for descriptions of the exercises and the cognitive skills trained by each). However, transfer to these domains was not formally tested and remains an open area for further research.

Our study has several other limitations. First, the sample size was quite small and warrants replication. Second, the results did not identify cognitive skills that are essential to bookkeeping performance or factors (e.g., age, experience, and compensation) that may predict response to training. Third, the subjects were a highly-educated, young, and relatively-restricted university-based volunteer cohort, unlike the heterogeneous workforce. Subjects were also highly motivated to complete the training (and did so) for secondary gains. Whether workers in a busy work environment facing multiple time- and productivity- pressures would respond similarly is debatable, and the dollar value of CCT to organizations is unknown.

To that end, we have recently reported negative results from a larger trial of CCT in organizational settings (Borness et al., 2013). This trial was based on short (15–20 min) self-administered sessions, whereas the current study provided 60-min group-based, supervised sessions. Supervision and support may therefore be crucial for training success (Salas and Burke, 2002; Sandford, 2003; Medalia and Richardson, 2005), and was associated with greater effects in healthy older adults (Kelly et al., 2014). Other differences between these two studies, most notably the use of distinct CCT programs and choice of productivity-related endpoints emphasize the need for more specific protocols in this area.

Improving workforce cognition along the life span may be a key factor in maintaining economic prosperity in the context of ageing populations and growing weight of cognitively demanding tasks in the contemporary workplace. Though limited in scope, our laboratory-based study suggests that supervised CCT can help improve performance on a work-related task such as bookkeeping. Field-based research in workplace settings is now required to test the idea that CCT can boost workers' cognitive abilities and translate to enhanced real-world occupational outcomes such as work productivity.

### Conflict of interest statement

Dr. Valenzuela received funding from the Brain Department LLC as well as in-kind support from BrainTrain Inc for projects unrelated to this study.
