# Supplementary material for: Multi-domain computerized cognitive training program improves performance of bookkeeping tasks: a matched-sampling active-controlled trial
Source: Front Psychol. 2014 Jul 28;5:794. doi: 10.3389/fpsyg.2014.00794 (PMC4112995; doi:10.3389/fpsyg.2014.00794)
Supplement: Supplementary file 1 [file DataSheet1.DOCX]

***Supplementary Material***

**Multi-domain computerized cognitive training program improves performance of bookkeeping tasks:  a matched-sampling active-controlled trial**

**Amit Lampit^1,2^*, Claus Ebster^2^, Michael Valenzuela^1^**

^1^Regenerative Neuroscience Group, Brain and Mind Research Institute, University of Sydney, Sydney, NSW, Australia. ^2^Lauder Business School, Vienna, Austria.

^3^Department of Marketing, University of Vienna, Vienna, Austria.

*** Correspondence:** Amit Lampit, Regenerative Neuroscience Group, Brain and Mind Research Institute, University Of Sydney, 94 Mallett St, Camperdown, NSW, 2050, Australia.

amit.lampit@sydney.edu.au

1. **Bookkeeping Transfer Task**

## Journal of Transactions

## Instructions Sheet

## Blank General Ledger

## Answer Template for the Transactions in 1.1

1. **Exercise Descriptions**

## Conceptual Discrimination (The Ugly Duckling) Purpose of Program:

The Conceptual Discrimination program trains the conceptual abilities to perceive, classify, and recognize different images based on an underlying rule of logic. The player is shown a series of boxes, and must choose the box that is different from all the others in some way.

This program trains the following skills:

- Conceptual Reasoning
- Visual Perception
- Central Processing Speed
- Working Memory

## Logical Sequences (Happy Trails) Code Name: CON-2

The Logical Sequences program trains conceptual reasoning and visuospatial sequencing. It helps to develop and train a person’s ability to remember and find sequential patterns of different types of target stimuli. These patterns may be comprised of numbers, letters or images. The stimuli may be both spoken and shown. In some cases only visual images will be presented. The player is then shown a grid of letters, numbers and/or images. The goal is to find and click on the targets in the correct sequence given.

Note that it is not recommended that both the Audio Cues and Visual Cues be turned off at the same time, as this essentially turns the task into a guessing game.

This program trains the following skills:

- Conceptual Reasoning
- Visuospatial Sequencing
- Selective Attention
- Visual Processing Speed
- Working Memory

## Numeric Skills (Total Recall)

The Numeric Skills program trains an individual’s conceptual reasoning and visuospatial classification skills. These skills are trained in a series of exercises that require detailed visual perception, working memory and classification abilities. In most of the exercises the players are shown a variety of images and they must count the designated target stimuli on the screen. Also, players may be shown simple mathematical problems and asked for the answers. The math problems may be shown in a standard format or they may be shown graphically, using boxes of different images to represent the numbers.

This program trains the following skills:

- Conceptual Reasoning
- Visuospatial Classification
- General Attention
- Visual Processing Speed
- Working Memory

## Pattern Display Match (Domino Dynamite)

The Pattern Display Match program trains visual scanning and short-term memory of non-verbal patterened stimuli. The player is shown two patterns either side-by-side or above one another, and the player is required to look them over and tell if they are the same or different.

This program trains the following skills:

- Conceptual Reasoning
- Focused Attention
- Visual Perception
- Visual Processing Speed

## Size Discrimination (Tower Power)

The Size Discrimination program trains visual scanning and immediate visual memory. The player is shown a grid of objects, and he must click on the target objects in order according to their size.

Note that it is not recommended that both the Audio Cues and Visual Cues be turned off at the same time, as this essentially turns the program into a guessing game.

This program trains the following skills:

- Conceptual Reasoning
- Fine Motor Control
- General Attention
- Visuospatial Classification
- Visuospatial Sequencing
- Visual Perception
- Visual Tracking

## Symbolic Display Match (Max's Match)

The Symbolic Display Match program helps develop a complex conceptual reasoning. Individuals will develop their central processing speed and ability to identify, discriminate, and classify visual images based on the rule given. The player is required to select targets and place them in the appropriate box based on various rules.

This program trains the following skills:

- Conceptual Reasoning
- Central Processing Speed
- Visuospatial Classification
- General Attention

## Visual Pattern Recognition (What's Next?)

The Visual Pattern Recognition program trains the ability to recognize patterns. The player is shown a sequence of letters, numbers and/or images. The player is required to find the missing part of the sequence from among several choices.

Note that it is not recommended that both the Audio Cues and Visual Cues be turned off at the same time, as this essentially turns the program into a guessing game.

This program trains the following skills:

- Central Processing Speed
- Conceptual Reasoning
- General Attention
- Visuospatial Classification
- Visuospatial Sequencing
- Visual Perception

## Numeric Classification (Bits and Pieces)

The Numeric Classification program trains visuospatial classification and working memory. Players will see a number of images displayed on the screen. These images will vary by color, shape, size, and category. The player will be required to follow the rules given at the beginning of each stage. The target images must be accurately classified and counted based on the rule given. Sometimes, the targets will be visually distracting or blink on and off. The player must hold the rule in their mind and perform mental math in order to successfully pass each stage.

This program trains the following skills:

- Visuospatial Classification
- Working Memory
- Conceptual Reasoning
- Central Processing Speed
- Visual Perception
- General Attention

## Numeric Combinations (Match Maker)

The Numeric Combinations program trains visual perception and selective attention. Players will usually see a set of images at the top of the screen. Next, they will have to find the box that contains these images based on the rule given. These images will vary by color, shape, size, and category. The target images must be accurately classified and identified based on the rule given. Sometimes, the targets will blink on and off. The player must hold the rule in their mind and accurately identify the target images in the various boxes.

This program trains the following skills:

- Visual Perception
- Selective Attention
- Conceptual Reasoning
- Alternating Attention
- Immediate Memory

## Numeric Discrimination (City Lights)

The Numeric Discrimination program trains conceptual reasoning and visuospatial classification. Players will usually see boxes containing either images or numbers. They will have to find the box that contains the targets based on the rule given. These images will vary by color, shape, size, and category. The target images must be accurately classified and often counted. Sometimes, the targets will blink on and off. The player must hold the rule in their mind and use their conceptual reasoning skills in order to make accurate choices and pass the stages.

This program trains the following skills:

- Conceptual Reasoning
- Visuospatial Classification
- Working Memory
- General Attention

## Numeric Distinctions (Counting Critters)

The Numeric Distinctions program trains visuospatial sequencing and conceptual reasoning. Players will see numbers, images and objects of various lengths. The images will often vary by color, size, and the number of them displayed on the screen. The player will be required to follow the rules given at the beginning of each stage. The target images must be accurately identified and counted based on the rule given. Sometimes, the targets will blink on and off. The player will be required to use their visual perception abilities and working memory abilities to make judgements and decisions about the various targets presented in order to successfully pass each stage.

This program trains the following skills:

- Visuospatial Sequencing
- Conceptual Reasoning
- Visuospatial Classification
- Working Memory
- Immediate Memory
- General Attention
- Visual Perception

## Numeric Recall (Happy Hunter)

The Numeric Recall program trains immediate memory and focused attention. Players will be presented numbers in sets and also sequentially. Most of the exercises will require them to remember the numbers presented to them based on the rule given. The numbers will often vary by color and size. As the exercises progress, it is required that the player remember longer and more complex numeric sequences. Visuospatial memory is also trained in this program. At the highest levels, conceptual reasoning skills will be necessary to identify the most likely numbers that need to be remembered based on key characteristics given in the rule.

This program trains the following skills:

- Immediate Memory
- Focused Attention
- Working Memory
- Visuospatial Sequencing
- Conceptual Reasoning
